# The Challenges of Microbial Control of Mosquito-Borne Diseases Due to the Gut Microbiome

**DOI:** 10.3389/fgene.2020.504354

**Published:** 2020-10-07

**Authors:** Daniel P. Dacey, Frédéric J. J. Chain

**Affiliations:** Department of Biological Sciences, University of Massachusetts Lowell, Lowell, MA, United States

**Keywords:** 16S, *Asaia*, Bti, *Chromobacterium*, microbiome, mosquito, pathogen, *Wolbachia*

## Abstract

Mosquitoes are one of the deadliest animals on earth because of their ability to transmit a wide range of human pathogens. Traditional mosquito control methods use chemical insecticides, but with dwindling long-term effectiveness and negative effects on the environment, microbial forms of control have become common alternatives. The insecticide *Bacillus thuringiensis* subspecies *israelensis* (Bti) is the most popular of these alternatives, although it can also have direct effects on lowering environmental biodiversity and indirect effects on food-web relationships in the ecosystems where it is deployed. In addition, microbial control agents that impede pathogen development or transmission from mosquito to human are under investigation, including *Wolbachia* and *Asaia*, but unexpected interactions with mosquito gut bacteria can hinder their effectiveness. Improved characterization of mosquito gut bacterial communities is needed to determine the taxa that interfere with microbial controls and their effectiveness in wild populations. This mini-review briefly discusses relationships between mosquito gut bacteria and microbial forms of control, and the challenges in ensuring their success.

## Introduction

Mosquitoes are major disease vectors causing more than 700,000 deaths and millions of new infections every year (World Health Organization, 2017). Chemical insecticides remain the most widely used form of mosquito control, but with a rise in resistant mosquito populations (Dai et al., 2015; Moyes et al., 2017; Li et al., 2018), contribution to population declines of off-target insects including bees, ladybugs, and wasps (Gossett, 2018), and dangers to human health (van den Berg et al., 2015), alternative approaches remain necessary. Since its commercialization in 1938, the insecticide *Bacillus thuringiensis* subspecies *israelensis* (Bti) has been a popular alternative to chemical insecticides and is the most common microbial form for controlling mosquitoes today (Sanahuja et al., 2011; Zhang et al., 2017). Though Bti largely overcomes the specific pitfalls of chemical treatments (Sanahuja et al., 2011), concerns remain over the direct effects of toxicity to off-target organisms that may lower environmental biodiversity, and the potentially detrimental indirect effects on both terrestrial and aquatic food-webs (as reviewed by Bruhl et al., 2020). With the ongoing widespread use of chemical insecticides and concerns lingering around long-term Bti use, other microbes have come to the forefront as potential control agents for mosquito-borne diseases.

Many proposed microbial control agents are symbiotic bacteria that have been exploited for their abilities to interfere with human pathogen viability within mosquitoes or reduce adult host lifespan and reproductive abilities (Minard et al., 2013a; Huang et al., 2020). These differ from insecticides, chemical or microbial, which kill the vectors of disease transmission. Microbial control agents can be native endosymbionts of a target mosquito species that are exploited for human pathogen control, such as the *Wolbachia w*Pip strain in *Culex pipiens* (Glaser and Meola, 2010). They can also be endosymbionts from an insect different from the target mosquito species that are transfected into a target host, such as the *w*Mel strain of *Wolbachia* with antiviral properties taken from *Drosophila melanogaster* and transfected into *Aedes aegypti* for yellow fever virus reduction (van den Hurk et al., 2012). Such microbial control agents may be genetically manipulated in the lab to produce strains that allow for improved host inoculation, bacterial colonization, and vertical and horizontal transmission.

Given the key roles that the host gut microbiome plays in the development of a functioning immune system (as reviewed by Hooper et al., 2012), the triggering of mosquito immune responses to modulate vector competence to human pathogens, and the production of bacterial metabolites that inhibit human pathogens directly (Ramirez et al., 2012, 2014; Chu and Mazmanian, 2013), one can anticipate that exposure to microbial controls influence mosquitoes through a complex interplay between the microbiome and the immune response, where different contexts can affect control success. For example, the mosquito gut microbiome has shown to be important for some microbial forms of control to be effective (Caccia et al., 2016), while at other times having little influence (Ramirez et al., 2014) or proving to be a hindrance to their efficacy (Patil et al., 2013; Hughes et al., 2014; Rossi et al., 2015; Zink et al., 2015).

While microbial interactions within a mosquito can have important physiological repercussions on the host and on their susceptibility to pathogens (Guégan et al., 2018), the conditions under which they affect microbial control effectiveness are mostly unknown. Furthermore, as lab populations of mosquitoes can have distinct microbiomes compared to their wild counterparts (Hegde et al., 2018), such microbial interactions are difficult to predict when bringing a method of mosquito control from lab to field. While both direct and indirect effects of the mosquito gut microbiota can result in either positive or negative interactions with microbial forms of control, as reviewed elsewhere (Guégan et al., 2018; Tetreau, 2018), this mini-review focuses on the pros and cons of Bti and three candidate microbial control agents, the impact of gut bacteria on their efficacy, and some of the current limitations in characterizing gut microbiota of wild mosquito populations.

## Bti

Bti produces four protoxins (Cry4A, Cry4B, Cry11A, and Cyt1A) as three distinct crystals that become lethal once ingested by mosquito larvae. Enzymes present in the larval gut activate these protoxins to a toxic state, form pores in the gut epithelial membrane, and lead to larval death. In certain environmental conditions, Bti toxins can persist for months after application, possibly exposing mosquito larvae to low concentrations of toxin for a prolonged period that could favor resistance development, but with the combination of all four toxins this is not a likely outcome (Bruhl et al., 2020). Bti tolerance in wild mosquito populations has only been reported twice, in a population of *C. pipiens* in Syracuse, New York (Paul et al., 2005) and an *Aedes rusticus* population in the French Rhône-Alpes (Boyer et al., 2012), with no further published reports indicating sustained selected resistance in these areas. Nevertheless, several investigations have been carried out to understand the basis of potential Bti tolerance development using lab-bred mosquitoes (Paris et al., 2012; Tetreau et al., 2013; Stalinski et al., 2014) with the causal mechanisms likely involving individual target-site modifications for each toxin.

There is some evidence, however, showing that the action of Bti is affected by mosquito gut bacteria. While there are conflicting results about the role that the midgut microbial community plays in the insecticidal activity and toxicity of Bt in lepidopterans (Broderick et al., 2006, 2009; Johnston and Crickmore, 2009; Raymond et al., 2010; Caccia et al., 2016), the effectiveness of Bti against mosquitoes has been shown to be enhanced by decreasing gut bacterial diversity before introduction of the insecticide. Populations of *Anopheles stephensi* with sterile guts show increased susceptibility to Bti compared to those colonized with bacteria, wherein the microaerophilic conditions of the normal larval gut might promote the degradation of Bti toxins via bacteria using them as a nitrogen source (Patil et al., 2013). Despite this hindrance by the gut bacterial community on Bti efficacy, the specific bacterial isolate that is responsible has yet to be identified. Conversely, Bti itself alters the microbiome of *A. aegypti* larvae upon introduction, resulting in lower bacterial diversity in those larvae most tolerant to the insecticide when compared to individuals unexposed to Bti (Tetreau et al., 2018). The presence of Bti might therefore negatively impact the protective action of symbiotic bacteria, for example by reducing the bacterium *Acinetobacter*, which may have antiviral properties (Lee et al., 2009) as it highly associates with *A. aegypti* (David et al., 2016) and *Aedes albopictus* (Minard et al., 2013b) in organs where viruses replicate (Crotti et al., 2009; Zouache et al., 2009). Though Bti is still considered a safer alternative to chemical insecticides, it is unclear if its continued release into the environment will result in persistence of mosquito populations with altered microbiomes that reduce the effectiveness of Bti or have reduced abilities to protect against other pathogens. Perhaps more concerning is the increasing evidence of both direct and indirect effects on off-target organisms that can lower ecosystem biodiversity and alter trophic food webs, highlighting the need for alternative mosquito control agents.

## Wolbachia

*Wolbachia* is a genus of obligate intracellular bacteria found in many mosquito species. These bacteria can transmit vertically within an invertebrate population and horizontally within and across populations (Hughes et al., 2014). *Wolbachia* can also alter the fitness of infected insects (e.g., increase adult mortality), inhabit insect reproductive organs to cause cytoplasmic incompatibility between sperm and egg, and inhabit the insect gut, which is a key environment for the replication and maturation of many human pathogens (Ricci et al., 2012; Dorigatti et al., 2018). Some natural strains of *Wolbachia* display inhibitory mechanisms against human pathogens such as arboviruses (Caragata et al., 2019), for example by diminishing the viral load of West Nile in *C. pipiens* and *Culex restuans* (Novakova et al., 2017). This makes *Wolbachia* a good microbial control agent candidate by means of targeting the pathogen as opposed to the mosquito itself, reducing off-target effects that negatively affect environmental biodiversity and food-web networks. In addition, *Wolbachia* has been found to have no negative impacts on the efficacy of Bti (Endersby and Hoffmann, 2013), therefore possibly allowing both control methods to be used simultaneously. While *Wolbachia* is widespread throughout many wild mosquito species of *Aedes*, *Culex*, *Coquillettidia*, and *Mansonia* (Ricci et al., 2012), lab strains of *Wolbachia* have been genetically manipulated for manual transinfection in mosquito species that do not typically host *Wolbachia*. *A. aegypti* is one of these species not naturally infected with *Wolbachia* but the introduction of this bacterium to the mosquito’s gut has inhibited host infection by *Plasmodium* parasites and dengue, chikungunya, West Nile, and yellow fever viruses (Moreira et al., 2009; Bourtzis et al., 2014; Jeffries and Walker, 2015). Viral inhibition by *Wolbachia* might be achieved through competition for cellular resources (Moreira et al., 2009) or by increasing expression of NADPH oxidase, which up-regulates reactive oxygen species and leads to host immune pathway activation (Pan et al., 2012). An increase in adult mortality due to *Wolbachia* infection may also contribute to lessening mosquito-borne virus transmission to humans, with mosquitoes dying younger thus not giving a virus enough time to replicate and migrate to the salivary glands for human inoculation (McMeniman et al., 2009). *Plasmodium* infection in *Anopheles* mosquitoes has also been suppressed by the introduction of *Wolbachia* lab strains (Bian et al., 2013), plausibly by the same mechanisms as for *A. aegypti* virus inhibition (Moreira et al., 2009; Pan et al., 2012).

The establishment of *Wolbachia* is, however, itself inhibited in the presence of certain commensal gut bacteria, limiting its effectiveness as a way to control the spread of mosquito-borne human pathogens. For example, the effectiveness of *Wolbachia* depends on the absence of *Asaia*, another prevalent bacterium naturally found in the guts of many mosquito species. *Asaia* and *Wolbachia* often compete with one another for resources that can lead to mutual exclusion, with wild strains of *Asaia* shown to inhibit the establishment of *Wolbachia* biologics via negative interference competition in *Anopheles* and *A. aegypti* mosquitoes (Hughes et al., 2014; Rossi et al., 2015). Likewise, the presence of native *Serratia* in the mosquito gut is also negatively correlated with the presence of *Wolbachia*, thus possibly impeding the establishment of *Wolbachia* (Zink et al., 2015). On the other hand, *Wolbachia*, which may suppress pathogen transmission to humans by infecting mosquito reproductive organs to cause cytoplasmic incompatibility, has not shown to have a major impact on the gut bacterial diversity of lab reared *A. aegypti* or *A. stephensi* (Chen et al., 2016; Audsley et al., 2017) or wild *A. aegypti* mosquitoes (Audsley et al., 2018), questioning its ability to impact host fitness in this regard. The mechanisms by which inhibitory gut bacteria may interfere with *Wolbachia* are not known, but their continued presence is possibly one of the reasons why some mosquito populations are unable to be stably infected with strains of *Wolbachia* (Hughes et al., 2014; Rossi et al., 2015; Zink et al., 2015) compared to others that are successful (Garcia et al., 2019; Nazni et al., 2019). This variability should raise concerns about *Wolbachia* as a universally effective microbial control agent against mosquitoes.

## Asaia

*Asaia* is another bacterial genus being considered for control applications for its ability to infect multiple host organs via mosquito hemolymph and its ability to establish itself in numerous natural mosquito populations via vertical and horizontal modes of transmission (Rami et al., 2018). Several important mosquitoes commonly host *Asaia* including *Anopheles gambiae*, *A. stephensi*, *A. albopictus*, *A. aegypti*, and most *C. pipiens* populations (Wilke and Marrelli, 2015). Some wild strains of *Asaia* have the ability to secrete proteins that are toxic to *Plasmodium* and limit the parasite’s reproduction in *Anopheles* vectors (Favia et al., 2007; Bongio and Lampe, 2015; Cappelli et al., 2019).

Unfortunately, common DNA sequencing methods are often unable to differentiate between different *Asaia* species within a bacterial community, limiting our ability to exploit the beneficial anti-pathogenic properties of a particular species within this genus. The identification of the specific *Asaia* strains that reduce mosquito competence of a human pathogen (e.g., by hindering *Plasmodium* reproduction) will enable development of microbial control agents with higher success rates against disease transmission, but might require a combination of sequencing techniques and biochemical tests for species level resolution and isolation (Rami et al., 2018). However, because *Asaia* competes with *Wolbachia* in the gut of target mosquitoes, there will likely be a loss of anti-pathogen activity in certain circumstances if exclusion of *Wolbachia* is unavoidable (Rossi et al., 2015), weakening its ability to prevent mosquito-borne disease transmission to humans since *Wolbachia* is widespread. As both *Asaia* and *Wolbachia* show potential as candidates for mosquito-borne disease control, it is important to keep in mind their exclusionary relationship, calling for methods of selectively inhibiting one or the other for example by genetic engineering.

## Chromobacterium

A bacterial symbiont recently isolated from an *Aedes* population and being considered for mosquito-borne disease control is *Chromobacterium* sp. Panama (Csp_P). This bacterium can prevent dengue virus and *Plasmodium falciparum* infection in lab-reared *A. aegypti* and *A. gambiae*, respectively, by rapidly colonizing the mosquito gut, prompting expression of host immune factors, and maintaining an effective level of host innate immunity (Ramirez et al., 2014). Csp_P also secretes an aminopeptidase that degrades the envelope protein of dengue virus that is required for attachment to host cells (Saraiva et al., 2018a) and romidepsin, a histone deacetylase inhibitor, that suppresses *Plasmodium* development in *A. gambiae* mosquitoes (Saraiva et al., 2018b). The entomopathogenic activity of Csp_P seems to be independent of the presence of a gut bacterial community, as rapid mortality is seen in lab-reared *A. aegypti* and *A. gambiae* with both aseptic and bacteria inhabited guts (Ramirez et al., 2014). As lab-raised mosquitoes have distinctly different gut bacterial communities than wild mosquitoes (Hegde et al., 2018), field studies are still needed to determine if Csp_P has any significant microbial interactions in wild target populations, and if it is able to be established in such populations with continued anti-malarial and anti-viral properties.

There are several other bacteria besides the ones mentioned that can inhibit transmission of human pathogens like La Crosse virus, dengue virus, and *Plasmodium* in mosquito vectors including *Anopheles*, *Aedes*, and *Culex*, but their lack of wide-spread presence throughout mosquito populations and the lack of knowledge regarding interference by other microbes has not been well established, limiting their potential at this point (Cirimotich et al., 2011; Joyce et al., 2011; Ramirez et al., 2012, 2014; Bando et al., 2013; Bahia et al., 2014; Dennison et al., 2016). Another challenge is that some control agents are species-specific and can even enhance vectorial capacity of other human pathogens, which is a problem for implementation in nature.

## Inadvertent Effects of Microbial Control in Wild Populations

Microbial control agent candidates that display anti-transmission properties of a human pathogen in one mosquito species have sometimes been found to increase mosquito susceptibility to infection of the same or different human pathogen in another species. For example, although *Wolbachia* strains can suppress *Plasmodium* infection in *Aedes* and *Anopheles*, they can actually increase *Plasmodium* susceptibility in *C. pipiens* (Zele et al., 2014). Furthermore, while *Wolbachia* strains might be effective against *Plasmodium* in *A. aegypti*, they can increase susceptibility to dengue virus infection in these mosquitoes (King et al., 2018). Similarly, Bti-exposed lab strains of *A. aegypti* larvae bred to have low levels of resistance to Bti display a higher susceptibility to dengue virus as adults than individuals of this strain not exposed to the insecticide (Moltini-Conclois et al., 2018). Another promising microbial candidate displaying anti-malarial properties in *Anopheles* vectors is *Serratia* (Bando et al., 2013; Wang et al., 2017), but the presence of the bacterium promotes dengue and Chikungunya viral infections in *Aedes* mosquitoes (Apte-Deshpande et al., 2012, 2014; Wu et al., 2019). The introduction of a microbial control agent in the wild to manage the spread of one group of human pathogens must therefore not inadvertently get transmitted to the environment or to other mosquito species, or otherwise risk an inadvertent mosquito-borne disease outbreak.

## Challenges in Characterizing Mosquito Gut Bacterial Communities

To improve the chances of deploying successful microbial control agents to control mosquito-borne diseases, there needs to be concerted efforts to characterize the community of bacteria harbored by the guts of target mosquito populations. A major challenge is our ability to accurately assess the composition and diversity of microbial gut communities. These problems relate to the experimental conditions and methods used that might bias the distribution of bacteria observed, as well as the technical limitations of current sequencing and bioinformatics approaches.

The bacterial community composition of the mosquito gut is influenced by the water condition of breeding sites (Gimonneau et al., 2014; Muturi et al., 2018; Duguma et al., 2019), the developmental stage of the mosquito (Duguma et al., 2015; Receveur et al., 2018), and the mosquito species (Duguma et al., 2015; Muturi et al., 2016, 2017; Hegde et al., 2018; Thongsripong et al., 2018). The degree of influence that environmental microbes have on the colonization, succession, and temporal changes of bacteria in the mosquito gut remain unclear, but this is important to determine the long-term impact of microbials that alter microbiomes. So far, comparisons of gut bacteria among field-collected mosquito populations have revealed relatively little diversity at the phylum and family levels (Boissiere et al., 2012; Osei-Poku et al., 2012; Thongsripong et al., 2018). However, at lower levels of taxonomic classification gut bacterial diversity between mosquitoes increases significantly (Boissiere et al., 2012), some reports claiming up to a 90% difference in operational taxonomic units between two individual mosquitoes (Wang et al., 2011; Osei-Poku et al., 2012; Rosso et al., 2018). The inherent variation in biological and environmental factors affecting the mosquito microbiota continues to present challenges in predicting potential gut bacterial interactions with microbial control agents in nature.

While technological advances have enabled thorough investigations into the characterization of microbiomes, there still remains technical challenges to identify species and compare results across studies. Gut microbial communities are typically characterized using 16S ribosomal RNA (16S rRNA) gene sequencing, but inconsistencies in species profiling exist due to variations in experimental designs and analytical methods that introduce biases, preventing direct comparisons of microbial communities across studies (as reviewed by Pollock et al., 2018). For example, it has been shown that using different reference databases to classify sequences from the same dataset can result in substantial taxonomic differences for bacteria identified at low abundance (López-García et al., 2018). Furthermore, the method employed for DNA extraction and the choice of target region within the 16S sequence also both affect the taxonomic recovery of microbes in general (Teng et al., 2018).

Some of the limitations of 16S sequencing can be addressed using whole metagenome sequencing because of the information acquired from other regions in bacterial genomes. While this can be more costly and challenging, metagenomics offers advantages over 16S sequencing in its ability to profile bacterial genes and to both detect and identify a greater diversity of bacterial species (Ranjan et al., 2016). For example, whole metagenomics has uncovered for the first time a link between wild *Anopheles albimanus* microbiomes and resistance to the organophosphate insecticide fenitrothion (Dada et al., 2018). The combination of 16S and metagenomic sequencing has also been used to assess the influence of an *Anopheles* immune factor on the microbiota, revealing immune factor APL1 to have homeostatic control on the abundance of specific *Cedecea* and *Klebsiella* species in the gut (Mitri et al., 2020). Similarly, the Caudal gene in *D. melanogaster* has a direct impact on the gut bacterial community but at a more general level, as this gene is responsible for repressing expression of antimicrobial peptide genes in the digestive tract (Ryu et al., 2008). Exploitation of mosquito immune responses to manipulate host gut bacterial makeup may, for example, aid in transinfection success of microbial control agents by depleting inhibitory strains or by promoting dominance of natural symbionts known to inhibit human pathogens. As mosquito-transmitted pathogens continue to spread globally, we need the adoption of standardized robust techniques and analysis methods in mosquito microbiome research relating to microbial control agent development to permit cross-study comparisons and for the advancement of validating promising microbial controls in the field. This will facilitate selection of microbial agents with higher success rates of disease control by avoiding unwanted interactions with the mosquito gut microbiome.

## Author Contributions

DD conceived, designed, and wrote the manuscript. FC critically reviewed the manuscript. Both authors approved the final version of the manuscript.

## Conflict of Interest

The authors declare that the research was conducted in the absence of any commercial or financial relationships that could be construed as a potential conflict of interest.

## References

[B1] Apte-Deshpande A. Paingankar M. Gokhale M. D. Deobagkar D. N. (2012). Serratia odorifera a midgut inhabitant of *Aedes aegypti* mosquito enhances its susceptibility to dengue-2 virus. *PLoS One* 7:e40401. 10.1371/journal.pone.0040401 22848375 PMC3407224

[B2] Apte-Deshpande A. D. Paingankar M. S. Gokhale M. D. Deobagkar D. N. (2014). Serratia odorifera mediated enhancement in susceptibility of *Aedes aegypti* for chikungunya virus. *Indian J. Med. Res.* 139 762–768. 25027087 PMC4140042

[B3] Audsley M. D. Seleznev A. Joubert D. A. Woolfit M. O’Neill S. L. McGraw E. A. (2018). Wolbachia infection alters the relative abundance of resident bacteria in adult *Aedes aegypti* mosquitoes, but not larvae. *Mol. Ecol.* 27 297–309. 10.1111/mec.14436 29165845

[B4] Audsley M. D. Ye Y. H. McGraw E. A. (2017). The microbiome composition of *Aedes aegypti* is not critical for Wolbachia-mediated inhibition of dengue virus. *PLoS Negl. Trop. Dis.* 11:e0005426. 10.1371/journal.pntd.0005426 28267749 PMC5357062

[B5] Bahia A. C. Dong Y. Blumberg B. J. Mlambo G. Tripathi A. BenMarzouk-Hidalgo O. J. (2014). Exploring Anopheles gut bacteria for Plasmodium blocking activity. *Environ. Microbiol.* 16 2980–2994. 10.1111/1462-2920.12381 24428613 PMC4099322

[B6] Bando H. Okado K. Guelbeogo W. M. Badolo A. Aonuma H. Nelson B. (2013). Intra-specific diversity of *Serratia marcescens* in Anopheles mosquito midgut defines Plasmodium transmission capacity. *Sci. Rep.* 3:1641. 10.1038/srep01641 23571408 PMC3622076

[B7] Bian G. Joshi D. Dong Y. Lu P. Zhou G. Pan X. (2013). Wolbachia invades *Anopheles stephensi* populations and induces refractoriness to Plasmodium infection. *Science* 340 748–751. 10.1126/science.1236192 23661760

[B8] Boissiere A. Tchioffo M. T. Bachar D. Abate L. Marie A. Nsango S. E. (2012). Midgut microbiota of the malaria mosquito vector Anopheles gambiae and interactions with *Plasmodium falciparum* infection. *PLoS Pathog.* 8:e1002742. 10.1371/journal.ppat.1002742 22693451 PMC3364955

[B9] Bongio N. J. Lampe D. J. (2015). Inhibition of Plasmodium berghei development in mosquitoes by effector proteins secreted from Asaia sp. Bacteria using a novel native secretion signal. *PLoS One* 10:e0143541 10.1371/journal.pone.0143541 PMC4670117 26636338

[B10] Bourtzis K. Dobson S. L. Xi Z. Rasgon J. L. Calvitti M. Moreira L. A. (2014). Harnessing mosquito-Wolbachia symbiosis for vector and disease control. *Acta Trop.* 132 S150–S163. 10.1016/j.actatropica.2013.11.004 24252486

[B11] Boyer S. Paris M. Jego S. Lemperiere G. Ravanel P. (2012). Influence of insecticide *Bacillus thuringiensis* subsp. israelensis treatments on resistance and enzyme activities in *Aedes rusticus larvae (Diptera: Culicidae)*. *Biol. Control* 62 75–81. 10.1016/j.biocontrol.2012.02.001

[B12] Broderick N. A. Raffa K. F. Handelsman J. (2006). Midgut bacteria required for *Bacillus thuringiensis* insecticidal activity. *Proc. Natl. Acad. Sci. U.S.A.* 103 15196–15199. 10.1073/pnas.0604865103 17005725 PMC1622799

[B13] Broderick N. A. Robinson C. J. McMahon M. D. Holt J. Handelsman J. Raffa K. F. (2009). Contributions of gut bacteria to *Bacillus thuringiensis*-induced mortality vary across a range of Lepidoptera. *BMC Biol.* 7:11. 10.1186/1741-7007-7-11 19261175 PMC2653032

[B14] Bruhl C. A. Despres L. Fror O. Patil C. D. Poulin B. Tetreau G. (2020). Environmental and socioeconomic effects of mosquito control in Europe using the biocide *Bacillus thuringiensis* subsp. *israelensis (Bti)*. *Sci. Total Environ.* 724:137800. 10.1016/j.scitotenv.2020.137800 32249002

[B15] Caccia S. Di Lelio I. La Storia A. Marinelli A. Varricchio P. Franzetti E. (2016). Midgut microbiota and host immunocompetence underlie *Bacillus thuringiensis* killing mechanism. *Proc. Natl. Acad. Sci. U.S.A.* 113 9486–9491. 10.1073/pnas.1521741113 27506800 PMC5003288

[B16] Cappelli A. Damiani C. Mancini M. V. Valzano M. Rossi P. Serrao A. (2019). Asaia activates immune genes in mosquito eliciting an anti-plasmodium response: implications in malaria control. *Front. Genet.* 10:836 10.3389/fgene.2019.00836 PMC6774264 31608103

[B17] Caragata E. P. Tikhe C. V. Dimopoulos G. (2019). Curious entanglements: interactions between mosquitoes, their microbiota, and arboviruses. *Curr. Opin. Virol.* 37 26–36. 10.1016/j.coviro.2019.05.005 31176069 PMC6768729

[B18] Chen S. Zhao J. Joshi D. Xi Z. Norman B. Walker E. D. (2016). Persistent infection by wolbachia wAlbB has no effect on composition of the gut microbiota in adult female *Anopheles stephensi*. *Front. Microbiol.* 7:1485. 10.3389/fmicb.2016.01485 27708633 PMC5030273

[B19] Chu H. Mazmanian S. K. (2013). Innate immune recognition of the microbiota promotes host-microbial symbiosis. *Nat. Immunol.* 14 668–675. 10.1038/ni.2635 23778794 PMC4109969

[B20] Cirimotich C. M. Dong Y. Clayton A. M. Sandiford S. L. Souza-Neto J. A. Mulenga M. (2011). Natural microbe-mediated refractoriness to Plasmodium infection in Anopheles gambiae. *Science* 332 855–858. 10.1126/science.1201618 21566196 PMC4154605

[B21] Crotti E. Damiani C. Pajoro M. Gonella E. Rizzi A. Ricci I. (2009). Asaia, a versatile acetic acid bacterial symbiont, capable of cross-colonizing insects of phylogenetically distant genera and orders. *Environ. Microbiol.* 11 3252–3264. 10.1111/j.1462-2920.2009.02048.x 19735280

[B22] Dada N. Sheth M. Liebman K. Pinto J. Lenhart A. (2018). Whole metagenome sequencing reveals links between mosquito microbiota and insecticide resistance in malaria vectors. *Sci. Rep.* 8:2084 10.1038/s41598-018-20367-20364 PMC5794770 29391526

[B23] Dai Y. Huang X. Cheng P. Liu L. Wang H. Wang H. (2015). Development of insecticide resistance in malaria vector Anopheles sinensis populations from Shandong province in China. *Malar. J.* 14:62 10.1186/s12936-015-0592-598 PMC4338830 25880316

[B24] David M. R. Santos L. M. B. Vicente A. C. P. Maciel-de-Freitas R. (2016). Effects of environment, dietary regime and ageing on the dengue vector microbiota: evidence of a core microbiota throughout *Aedes aegypti* lifespan. *Memórias do Instituto Oswaldo Cruz* 111 577–587. 10.1590/0074-02760160238 27580348 PMC5027870

[B25] Dennison N. J. Saraiva R. G. Cirimotich C. M. Mlambo G. Mongodin E. F. Dimopoulos G. (2016). Functional genomic analyses of *Enterobacter*, Anopheles and Plasmodium reciprocal interactions that impact vector competence. *Malar. J.* 15:425 10.1186/s12936-016-1468-1462 PMC4994321 27549662

[B26] Dorigatti I. McCormack C. Nedjati-Gilani G. Ferguson N. M. (2018). using wolbachia for dengue control: insights from modelling. *Trends Parasitol.* 34 102–113. 10.1016/j.pt.2017.11.002 29183717 PMC5807169

[B27] Duguma D. Hall M. W. Rugman-Jones P. Stouthamer R. Terenius O. Neufeld J. D. (2015). Developmental succession of the microbiome of Culex mosquitoes. *BMC Microbiol.* 15:140 10.1186/s12866-015-0475-8 PMC4513620 26205080

[B28] Duguma D. Hall M. W. Smartt C. T. Debboun M. Neufeld J. D. (2019). Microbiota variations in *Culex nigripalpus* disease vector mosquito of West Nile virus and Saint Louis Encephalitis from different geographic origins. *PeerJ* 6:e6168. 10.7717/peerj.6168 30643680 PMC6330035

[B29] Endersby N. M. Hoffmann A. A. (2013). Effect of Wolbachia on insecticide susceptibility in lines of *Aedes aegypti*. *Bull. Entomol. Res.* 103:269. 10.1017/s0007485312000673 23149015

[B30] Favia G. Ricci I. Damiani C. Raddadi N. Crotti E. Marzorati M. (2007). Bacteria of the genus Asaia stably associate with *Anopheles stephensi*, an Asian malarial mosquito vector. *Proc. Natl. Acad. Sci. U.S.A.* 104 9047–9051. 10.1073/pnas.0610451104 17502606 PMC1885625

[B31] Garcia G. A. Sylvestre G. Aguiar R. da Costa G. B. Martins A. J. Lima J. B. P. (2019). Matching the genetics of released and local *Aedes aegypti* populations is critical to assure Wolbachia invasion. *PLoS Negl. Trop. Dis.* 13:e0007023 10.1371/journal.pntd.0007023 PMC6338382 30620733

[B32] Gimonneau G. Tchioffo M. T. Abate L. Boissiere A. Awono-Ambene P. H. Nsango S. E. (2014). Composition of Anopheles coluzzii and Anopheles gambiae microbiota from larval to adult stages. *Infect. Genet. Evol.* 28 715–724. 10.1016/j.meegid.2014.09.029 25283802

[B33] Glaser R. L. Meola M. A. (2010). The native Wolbachia endosymbionts of *Drosophila melanogaster* and *Culex quinquefasciatus* increase host resistance to West Nile virus infection. *PLoS One* 5:e11977. 10.1371/journal.pone.0011977 20700535 PMC2916829

[B34] Gossett D. R. (2018). *Pesticides and Herbicides (Environmental Impact). Encyclopedia of Environmental Issues, Revised Edition (Online Edition).* Pasadena, CA: Salem Press.

[B35] Guégan M. Zouache K. Démichel C. Minard G. Tran Van V. Potier P. (2018). The mosquito holobiont: fresh insight into mosquito-microbiota interactions. *Microbiome* 6:49 10.1186/s40168-018-0435-2 PMC5859429 29554951

[B36] Hegde S. Khanipov K. Albayrak L. Golovko G. Pimenova M. Saldana M. A. (2018). Microbiome interaction networks and community structure from laboratory -reared and field-collected *Aedes aegypti*, *Aedes albopictus*, and *Culex quinquefasciatus* mosquito vectors. *Front. Microbiol.* 9:2160. 10.3389/fmicb.2018.02160 30250462 PMC6140713

[B37] Hooper L. V. Littman D. R. Macpherson A. J. (2012). Interactions between the microbiota and the immune system. *Science* 336 1268–1273. 10.1126/science.1223490 22674334 PMC4420145

[B38] Huang W. Wang S. Jacobs-Lorena M. (2020). Use of microbiota to fight mosquito-borne disease. *Front. Genet.* 11:196. 10.3389/fgene.2020.00196 32211030 PMC7076131

[B39] Hughes G. L. Dodson B. L. Johnson R. M. Murdock C. C. Tsujimoto H. Suzuki Y. (2014). Native microbiome impedes vertical transmission of Wolbachia in Anopheles mosquitoes. *Proc. Natl. Acad. Sci. U.S.A.* 111 12498–12503. 10.1073/pnas.1408888111 25114252 PMC4151774

[B40] Jeffries C. L. Walker T. (2015). The potential use of wolbachia-based mosquito biocontrol strategies for Japanese encephalitis. *PLoS Negl. Trop. Dis.* 9:e0003576. 10.1371/journal.pntd.0003576 26086337 PMC4472807

[B41] Johnston P. R. Crickmore N. (2009). Gut bacteria are not required for the insecticidal activity of *Bacillus thuringiensis* toward the tobacco hornworm, *Manduca sexta*. *Appl. Environ. Microbiol.* 75 5094–5099. 10.1128/AEM.00966-9 19525273 PMC2725506

[B42] Joyce J. D. Nogueira J. R. Bales A. A. Pittman K. E. Anderson J. R. (2011). Interactions between La Crosse virus and bacteria isolated from the digestive tract of *Aedes albopictus* (Diptera: culicidae). *J. Med. Entomol.* 48 389–394. 10.1603/me09268 21485378

[B43] King J. G. Souto-Maior C. Sartori L. M. Maciel-de-Freitas R. Gomes M. G. M. (2018). Variation in Wolbachia effects on Aedes mosquitoes as a determinant of invasiveness and vectorial capacity. *Nat. Commun.* 9:1483 10.1038/s41467-018-03981-8 PMC5902584 29662096

[B44] Lee J. S. Lee K. C. Kim K. K. Hwang I. C. Jang C. Kim N. G. (2009). Acinetobacter antiviralis sp. nov., from Tobacco plant roots. *J. Microbiol. Biotechnol.* 19 250–256. 10.4014/jmb.0901.083 19349749

[B45] Li Y. Xu J. Zhong D. Zhang H. Yang W. Zhou G. (2018). Evidence for multiple-insecticide resistance in urban *Aedes albopictus* populations in southern China. *Parasit. Vectors* 11:4 10.1186/s13071-017-2581-y PMC5753460 29298700

[B46] López-García A. Pineda-Quiroga C. Atxaerandio R. Pérez A. Hernández I. García-Rodríguez A. (2018). Comparison of Mothur and QIIME for the analysis of rumen microbiota composition based on 16S rRNA amplicon sequences. *Front. Microbiol.* 9:3010. 10.3389/fmicb.2018.03010 30619117 PMC6300507

[B47] McMeniman C. J. Lane R. V. Cass B. N. Fong A. W. Sidhu M. Wang Y. F. (2009). Stable introduction of a life-shortening Wolbachia infection into the mosquito *Aedes aegypti*. *Science* 323 141–144. 10.1126/science.1165326 19119237

[B48] Minard G. Mavingui P. Moro C. V. (2013a). Diversity and function of bacterial microbiota in the mosquito holobiont. *Parasit. Vectors* 6:146. 10.1186/1756-3305-6-146 23688194 PMC3667145

[B49] Minard G. Tran F. H. Raharimalala F. N. Hellard E. Ravelonandro P. Mavingui P. (2013b). Prevalence, genomic and metabolic profiles of Acinetobacter and Asaia associated with field-caught *Aedes albopictus* from Madagascar. *FEMS Microbiol. Ecol.* 83 63–73. 10.1111/j.1574-6941.2012.01455.x 22808994

[B50] Mitri C. Bischoff E. Belda Cuesta E. Volant S. Ghozlane A. Eiglmeier K. (2020). Leucine-Rich immune factor APL1 is associated with specific modulation of enteric microbiome taxa in the asian malaria mosquito *Anopheles stephensi*. *Front. Microbiol.* 11:306. 10.3389/fmicb.2020.00306 32174902 PMC7054466

[B51] Moltini-Conclois I. Stalinski R. Tetreau G. Despres L. Lambrechts L. (2018). Larval exposure to the bacterial insecticide bti enhances dengue virus susceptibility of adult *Aedes aegypti* mosquitoes. *Insects* 9:193 10.3390/insects9040193 PMC6316598 30558130

[B52] Moreira L. A. Iturbe-Ormaetxe I. Jeffery J. A. Lu G. Pyke A. T. Hedges L. M. (2009). A Wolbachia symbiont in *Aedes aegypti* limits infection with dengue, Chikungunya, and Plasmodium. *Cell* 139 1268–1278. 10.1016/j.cell.2009.11.042 20064373

[B53] Moyes C. L. Vontas J. Martins A. J. Ng L. C. Koou S. Y. Dusfour I. (2017). Contemporary status of insecticide resistance in the major Aedes vectors of arboviruses infecting humans. *PLoS Negl. Trop. Dis.* 11:e0005625. 10.1371/journal.pntd.0005625 28727779 PMC5518996

[B54] Muturi E. J. Kim C.-H. Bara J. Bach E. M. Siddappaji M. H. (2016). *Culex pipiens* and Culex restuans mosquitoes harbor distinct microbiota dominated by few bacterial taxa. *Parasit. Vectors* 9:18. 10.1186/s13071-016-1299-6 26762514 PMC4712599

[B55] Muturi E. J. Lagos-Kutz D. Dunlap C. Ramirez J. L. Rooney A. P. Hartman G. L. (2018). Mosquito microbiota cluster by host sampling location. *Parasit. Vectors* 11:468. 10.1186/s13071-018-3036-9 30107817 PMC6092830

[B56] Muturi E. J. Ramirez J. L. Rooney A. P. Kim C.-H. (2017). Comparative analysis of gut microbiota of mosquito communities in central Illinois. *PLoS Negl. Trop. Dis.* 11:e0005377. 10.1371/journal.pntd.0005377 28245239 PMC5345876

[B57] Nazni W. A. Hoffmann A. A. NoorAfizah A. Cheong Y. L. Mancini M. V. Golding N. (2019). Establishment of wolbachia strain wAlbB in Malaysian populations of *Aedes aegypti* for dengue control. *Curr. Biol.* 29 4241–4248. e5. 10.1016/j.cub.2019.11.007 31761702 PMC6926472

[B58] Novakova E. Woodhams D. C. Rodriguez-Ruano S. M. Brucker R. M. Leff J. W. Maharaj A. (2017). Mosquito microbiome dynamics, a background for prevalence and seasonality of West Nile Virus. *Front. Microbiol.* 8:526. 10.3389/fmicb.2017.00526 28421042 PMC5378795

[B59] Osei-Poku J. Mbogo C. M. Palmer W. J. Jiggins F. M. (2012). Deep sequencing reveals extensive variation in the gut microbiota of wild mosquitoes from Kenya. *Mol. Ecol.* 21 5138–5150. 10.1111/j.1365-294X.2012.05759.x 22988916

[B60] Pan X. Zhou G. Wu J. Bian G. Lu P. Raikhel A. S. (2012). Wolbachia induces reactive oxygen species (ROS)-dependent activation of the Toll pathway to control dengue virus in the mosquito *Aedes aegypti*. *Proc. Natl. Acad. Sci. U.S.A.* 109 E23–E31. 10.1073/pnas.1116932108 22123956 PMC3252928

[B61] Paris M. Melodelima C. Coissac E. Tetreau G. Reynaud S. David J. P. (2012). Transcription profiling of resistance to Bti toxins in the mosquito *Aedes aegypti* using next-generation sequencing. *J. Invertebr. Pathol.* 109 201–208. 10.1016/j.jip.2011.11.004 22115744

[B62] Patil C. D. Borase H. P. Salunke B. K. Patil S. V. (2013). Alteration in *Bacillus thuringiensis* toxicity by curing gut flora: novel approach for mosquito resistance management. *Parasitol. Res.* 112 3283–3288. 10.1007/s00436-013-3507-z 23820604

[B63] Paul A. Harrington L. C. Zhang L. Scott J. G. (2005). Insecticide resistance in *Culex pipiens* from New York. *J. Am. Mosq. Control Assoc.* 21 305–309. 10.2987/8756-971x(2005)21[305:iricpf]2.0.co;2 16252522

[B64] Pollock J. Glendinning L. Wisedchanwet T. Watson M. (2018). The madness of microbiome: attempting to find consensus best practice for 16S microbiome studies. *Appl. Environ. Microbiol.* 84 e2627–17. 10.1128/AEM.02627-17 29427429 PMC5861821

[B65] Rami A. Raz A. Zakeri S. Dinparast Djadid N. (2018). Isolation and identification of Asaia sp. in *Anopheles* spp. mosquitoes collected from Iranian malaria settings: steps toward applying paratransgenic tools against malaria. *Parasit. Vectors* 11:367 10.1186/s13071-018-2955-59 PMC6022440 29950179

[B66] Ramirez J. L. Short S. M. Bahia A. C. Saraiva R. G. Dong Y. Kang S. (2014). Chromobacterium Csp_P reduces malaria and dengue infection in vector mosquitoes and has entomopathogenic and in vitro anti-pathogen activities. *PLoS Pathog.* 10:e1004398. 10.1371/journal.ppat.1004398 25340821 PMC4207801

[B67] Ramirez J. L. Souza-Neto J. Cosme R. T. Rovira J. Ortiz A. Pascale J. M. (2012). Reciprocal tripartite interactions between the *Aedes aegypti* midgut microbiota, innate immune system and dengue virus influences vector competence. *PLoS Negl. Trop. Dis.* 6:e1561. 10.1371/journal.pntd.0001561 22413032 PMC3295821

[B68] Ranjan R. Rani A. Metwally A. McGee H. S. Perkins D. L. (2016). Analysis of the microbiome: advantages of whole genome shotgun versus 16S amplicon sequencing. *Biochem. Biophys. Res. Commun.* 469 967–977. 10.1016/j.bbrc.2015.12.083 26718401 PMC4830092

[B69] Raymond B. Johnston P. R. Nielsen-LeRoux C. Lereclus D. Crickmore N. (2010). *Bacillus thuringiensis*: an impotent pathogen? *Trends Microbiol.* 18 189–194. 10.1016/j.tim.2010.02.006 20338765

[B70] Receveur J. P. Pechal J. L. Benbow M. E. Donato G. Rainey T. Wallace J. R. (2018). Changes in larval mosquito microbiota reveal non-target effects of insecticide treatments in hurricane-created habitats. *Microb. Ecol.* 76 719–728. 10.1007/s00248-018-1175-3 29549385

[B71] Ricci I. Damiani C. Capone A. DeFreece C. Rossi P. Favia G. (2012). Mosquito/microbiota interactions: from complex relationships to biotechnological perspectives. *Curr. Opin. Microbiol.* 15 278–284. 10.1016/j.mib.2012.03.004 22465193

[B72] Rossi P. Ricci I. Cappelli A. Damiani C. Ulissi U. Mancini M. V. (2015). Mutual exclusion of Asaia and Wolbachia in the reproductive organs of mosquito vectors. *Parasit. Vectors* 8:278. 10.1186/s13071-015-0888-0 PMC4445530 25981386

[B73] Rosso F. Tagliapietra V. Albanese D. Pindo M. Baldacchino F. Arnoldi D. (2018). Reduced diversity of gut microbiota in two Aedes mosquitoes species in areas of recent invasion. *Sci. Rep.* 8:16091. 10.1038/s41598-018-34640-z 30382151 PMC6208342

[B74] Ryu J. H. Kim S. H. Lee H. Y. Bai J. Y. Nam Y. D. Bae J. W. (2008). Innate immune homeostasis by the homeobox gene caudal and commensal-gut mutualism in *Drosophila*. *Science* 319 777–782. 10.1126/science.1149357 18218863

[B75] Sanahuja G. Banakar R. Twyman R. M. Capell T. Christou P. (2011). *Bacillus thuringiensis*: a century of research, development and commercial applications. *Plant Biotechnol. J.* 9 283–300. 10.1111/j.1467-7652.2011.00595.x 21375687

[B76] Saraiva R. G. Fang J. Kang S. Angleró-Rodríguez Y. I. Dong Y. Dimopoulos G. (2018a). Aminopeptidase secreted by Chromobacterium sp. Panama inhibits dengue virus infection by degrading the E protein. *PLoS Negl. Trop. Dis.* 12:e0006443 10.1371/journal.pntd.0006443 PMC5937796 29694346

[B77] Saraiva R. G. Huitt-Roehl C. R. Tripathi A. Cheng Y. Q. Bosch J. Townsend C. A. (2018b). Chromobacterium spp. mediate their anti-Plasmodium activity through secretion of the histone deacetylase inhibitor romidepsin. *Sci. Rep.* 8:6176 10.1038/s41598-018-24296-90 PMC5906607 29670144

[B78] Stalinski R. Tetreau G. Gaude T. Despres L. (2014). Pre-selecting resistance against individual Bti Cry toxins facilitates the development of resistance to the Bti toxins cocktail. *J. Invertebr. Pathol.* 119 50–53. 10.1016/j.jip.2014.04.002 24768915

[B79] Teng F. Darveekaran Nair S. S. Zhu P. Li S. Huang S. Li X. (2018). Impact of DNA extraction method and targeted 16S-rRNA hypervariable region on oral microbiota profiling. *Sci. Rep.* 8:16321 10.1038/s41598-018-34294-x PMC6218491 30397210

[B80] Tetreau G. (2018). Interaction between Insects, Toxins, and Bacteria: have we been wrong so far? *Toxins* 10:281 10.3390/toxins10070281 PMC6070883 29986377

[B81] Tetreau G. Grizard S. Patil C. D. Tran F.-H. Tran Van V. Stalinski R. (2018). Bacterial microbiota of *Aedes aegypti* mosquito larvae is altered by intoxication with *Bacillus thuringiensis* israelensis. *Parasit. Vectors* 11:121 10.1186/s13071-018-2741-48 PMC5834902 29499735

[B82] Tetreau G. Stalinski R. David J. P. Despres L. (2013). Increase in larval gut proteolytic activities and Bti resistance in the Dengue fever mosquito. *Arch. Insect. Biochem. Physiol.* 82 71–83. 10.1002/arch.21076 23192850

[B83] Thongsripong P. Chandler J. A. Green A. B. Kittayapong P. Wilcox B. A. Kapan D. D. (2018). Mosquito vector-associated microbiota: metabarcoding bacteria and eukaryotic symbionts across habitat types in Thailand endemic for dengue and other arthropod-borne diseases. *Ecol. Evol.* 8 1352–1368. 10.1002/ece3.3676 29375803 PMC5773340

[B84] van den Berg H. Yadav R. S. Zaim M. (2015). Setting international standards for the management of public health pesticides. *PLoS Med.* 12:e1001824. 10.1371/journal.pmed.1001824 25965399 PMC4428769

[B85] van den Hurk A. F. Hall-Mendelin S. Pyke A. T. Frentiu F. D. McElroy K. Day A. (2012). Impact of Wolbachia on infection with chikungunya and yellow fever viruses in the mosquito vector *Aedes aegypti*. *PLoS Negl. Trop. Dis.* 6:e1892. 10.1371/journal.pntd.0001892 23133693 PMC3486898

[B86] Wang S. Dos-Santos A. L. A. Huang W. Liu K. C. Oshaghi M. A. Wei G. (2017). Driving mosquito refractoriness to *Plasmodium falciparum* with engineered symbiotic bacteria. *Science* 357 1399–1402. 10.1126/science.aan5478 28963255 PMC9793889

[B87] Wang Y. Gilbreath T. M. III Kukutla P. Yan G. Xu J. (2011). Dynamic gut microbiome across life history of the malaria mosquito Anopheles gambiae in Kenya. *PLoS One* 6:e24767 10.1371/journal.pone.0024767 PMC3177825 21957459

[B88] Wilke A. B. Marrelli M. T. (2015). Paratransgenesis: a promising new strategy for mosquito vector control. *Parasit. Vectors* 8:342 10.1186/s13071-015-0959-52 PMC4489152 26104575

[B89] World Health Organization (2017). *Vector**-borne Diseases.* Available online at: https://www.who.int/en/news-room/fact-sheets/detail/vector-borne-diseases (accessed July 21, 2019).

[B90] Wu P. Sun P. Nie K. Zhu Y. Shi M. Xiao C. (2019). A gut commensal bacterium promotes mosquito permissiveness to arboviruses. *Cell Host Microbe* 25 101–112. e5. 10.1016/j.chom.2018.11.004. 30595552

[B91] Zele F. Nicot A. Berthomieu A. Weill M. Duron O. Rivero A. (2014). Wolbachia increases susceptibility to Plasmodium infection in a natural system. *Proc. Biol. Sci.* 281:20132837. 10.1098/rspb.2013.2837 24500167 PMC3924077

[B92] Zhang Q. Hua G. Adang M. J. (2017). Effects and mechanisms of *Bacillus thuringiensis* crystal toxins for mosquito larvae. *Insect Sci.* 24 714–729. 10.1111/1744-7917.12401 27628909

[B93] Zink S. D. Van Slyke G. A. Palumbo M. J. Kramer L. D. Ciota A. T. (2015). Exposure to west nile virus increases bacterial diversity and immune gene expression in *Culex pipiens*. *Viruses* 7 5619–5631. 10.3390/v7102886 26516902 PMC4632394

[B94] Zouache K. Voronin D. Tran-Van V. Mousson L. Failloux A. B. Mavingui P. (2009). Persistent Wolbachia and cultivable bacteria infection in the reproductive and somatic tissues of the mosquito vector *Aedes albopictus*. *PLoS One* 4:e6388 10.1371/journal.pone.0006388 PMC2712238 19633721

